# 

**Published:** 1898-08-27

**Authors:** 


**Notices of Births, Deaths, and Marriages are inserted in this column at a charge of 2s. 6d. for an announcement not exceeding four lines.**

### NOTICE TO CORRESPONDENTS.

**All MS., Letters, Books for Review, and other matters intended for the Editor should be addressed The Editor, "The Hospital," The Hospital Building, 28 & 29, Southampton Street, Strand, W.C.**

The Editor cannot undertake to return rejected MS., even when accompanied by stamped directed envelope.

**Notice to Advertisers and Subscribers.**

All Advertisements, Orders for Copies of The Hospital, and Business Communications should be addressed to **THE MANAGER, (Not to The Editor),** The Hospital, The Hospital Building, 28 & 29, Southampton Street, Strand, London, W.C.

The Cost of Subscription, post free, is as follows:—Per week, 2½d. per quarter, 2s. 8d.; per half-year, 5s. 3d.; per annum, 10s. 6d. Foreign:—Per annum, 15s. 2d.; payable, in all cases, in advance.

**NOTICE.—Vol. XXIII. of "The Hospital" (October, 1897, to March, 1898), in handsome cloth binding, is now on sale at the Office of this Journal, price 7s. 6d. Cases for binding the Numbers contained in this Volume, 1s. 6d. Index to Vol. XXIII., 2½d., post free.**

**The Monthly Part for September will be ready on September 1. Price 6d., by post 8d.**

### The Student of Medicine.

### ROYAL ARMY MEDICAL CORPS.

The following are among the examination papers set to the candidates for Army and Indian Medical Services at the recent examination:—

### Anatomy and Physiology.

1. Give the dissection necessary to expose the external pterygoid muscle, and describe the vessels and nerves in immediate relation to that muscle.
2. Describe the testicle and follow its duct from its commencement to its termination. Give, likewise, an account of the descent of the testicle, and explain the occurrence, in connection with this, of the occasional developmental defects which create a liability to the congenital and infantile forms of inguinal hernia.
3. Explain the part which is played by the vagus nerve in the function of respiration.
4. Give the minute structure of the submaxillary gland, and describe the changes which are observed to occur in the secretory cells as they pass from a quiescent or inactive condition into a state of activity. What is the active principle of saliva, and how does it act in the process of digestion?

### Medicine and Pathology.

1. Describe fully the symptoms and physical signs of aortic regurgitation, complicated with dilatation of the heart and general passive congestion, and say how you would treat it.
2. Enumerate, in tabular form, the various points which enable you to distinguish granular degeneration of the kidneys from acute nephritis.
3. What are the symptoms of subacute otitis media purulenta, complicated with implication of the facial nerve in the aqueduct of Fallopius?
4. Enumerate the enlargements of the liver, dividing them into (*a*) uniform and (*b*) irregular enlargements. Underline those which are tender, and put an asterisk opposite those in which the enlargement may be very great.

### Surgery.

(All four questions to be answered.)

1. Give the diagnosis, pathology, and treatment of new growths of the testicle.
2. What forms of congenital cyst and congenital fistula are met with in the regions of the head and neck? Describe their characters and mention any points of surgical importance in connection with their treatment.
3. In a case of gunshot wound traversing the middle of the thigh you apprehend, from the direction taken by the bullet, that the main artery may have been contused. Should this be so, what consequences may follow, and in what manner will you deal with them respectively?
4. Describe a case of chronic granular conjunctivitis. What are the causes, and what is the treatment, of the disease?

### Chemistry and Materia Medica.

1. What are the principal properties of the halogens, of sulphur, and of carbon? Mention the chief compounds of these elements with hydrogen and with oxygen respectively.
2. Explain the action of the more important chemical antiseptics and disinfectants.
3. What drugs act directly upon the heart? Name their preparations, and the dose of each for an adult.
4. What are the therapeutic use of arsenic and of antimony? Name the preparations of arsenic and of antimony, and state the proportion of the most important ingredient in each preparation.
5. Write prescriptions for the administration to adults of the following remedies in the cases mentioned with each, giving the full directions necessary: (*a*) Codeine (in diabetes); (*b*) quinine (in malarial fever); (*c*) ipecacuanha (in dysentery).

